# Hydrogen Absorption and Desorption Behavior on Aluminum-Coated Hot-Stamped Boron Steel during Hot Press Forming and Automotive Manufacturing Processes

**DOI:** 10.3390/ma14216730

**Published:** 2021-11-08

**Authors:** Hye-Jin Kim, Hyun-Yeong Jung, Seung-Pill Jung, Ji-Hee Son, Joo-Sik Hyun, Ju-Sung Kim

**Affiliations:** 1 Department of Automotive Application Engineering, Hyundai-Steel Company, 1480 Buckbusaneop-ro, Songak-Eup, Dangjin-Si 343-823, Korea; khj020911@snu.ac.kr (H.-J.K.); spjung@hyundai-steel.com (S.-P.J.); bjh7932@hyundai-steel.com (J.-H.S.); hjs410@hyundai-steel.com (J.-S.H.); sjkim2@hyundai-steel.com (J.-S.K.); 2 Department of Materials Science and Engineering &RIAM, Seoul National University, 1 Gwanak-ro, Soeul 08826, Korea; 3 Department of Material Science and Engineering, Korea University, 145 Anam-Ro, Seongbuk-Gu, Seoul 02841, Korea

**Keywords:** hot-stamped boron steel, thermal desorption spectroscopy, diffusible hydrogen

## Abstract

Our study mainly focused on diffusible hydrogen in aluminum–silicon-coated hot-stamped boron steel during a hot press forming process and in pre-treatment sequential lines of the automotive manufacturing process using a thermal desorption spectroscopy (TDS) technique. First, in the hot stamping procedure, as the soaking time increased in the heating furnace at a specific dew point when austenitizing, a high concentration of diffusible hydrogen was absorbed into the hot-stamped boron steel. Based on the TDS analysis of hydrogen absorbed from hot stamping, the activation energy value of hydrogen trapping in 1.8 GPa grade steel is lower than that of 1.5 GPa grade steel. This means that diffusible hydrogen can be more easily diffused into defective sites of the microstructure at a higher level of the tensile strength grade. Second, in sequential pre-treatment lines of the automotive manufacturing process, additional hydrogen did not flow into the surface, and an electro-deposition process, including a baking procedure, was effective in removing diffusible hydrogen, which was similar to the residual hydrogen of the as-received state (i.e., initial cold rolled blank). Based on these results, the hydrogen absorption was facilitated during hot press forming, but the hydrogen was sequentially desorbed during automotive sequential lines on aluminum-coated hot-stamped steel parts.

## 1. Introduction

Recently, hot stamping parts, which are extensively applied as ultra-high-strength steel, are in the spotlight due to their excellent formability and strength. The hot-stamped boron steel is a single-structure steel that has a microstructure composed of a hard martensite phase by heating a 600 MPa cold rolled grade steel sheet, which is composed of a ferrite phase and pearlite at a high-temperature austenizing temperature of 1173 K or higher, and rapidly cooling the steel sheet by using a metallic mold. In general, there are many advantages: it is possible to lighten the body weight and improve the collision performance with the application of low-alloy-based steels with an ultra-high-strength of above 1.0 GPa tensile strength. However, it is reported that martensite steel is highly vulnerable because of the phenomenon of a hydrogen delayed fracture, even though there is a small amount of hydrogen and residual stress in the steel [1,2,3]. A hydrogen delayed fracture is caused by the diffusion and accumulation of hydrogen that penetrates through the surface of the steel during the manufacturing process of the steel or in a corrosion environment when in usage of the vehicle. These hydrogen atoms can accumulate in the defective sites in the microstructure. These accumulated hydrogens induce the initiation and propagation of local cracks under stress states, and will eventually cause failure of the components. Hydrogen absorption from the surface to the internal microstructure is crucial. In previous studies, a substantial phenomenon and mechanism relevant to hydrogen absorption from the condition of the surface and its microstructural characteristics [3,4,5] has been reported. Therefore, a hydrogen diffusion behavior and the relationship of the hydrogen traps with the microstructure are indispensable research fields for the use of high-strength steels. As the application and demand for ultra-high-strength steel has constantly increased, the research for preventing hydrogen delay fractures has mainly emerged in order to ensure durability under harsh environments, along with its strength characteristics. In particular, in the case of hot stamping, it is reported that hydrogen atoms are decomposed from moisture due to a dew point phenomenon on the material surface. This phenomenon is caused due to a temperature difference of a heating furnace that heats the temperature to or maintains the temperature at 1173 K or more, and diffuses into aluminum plating and the austenite phase with a high solubility of hydrogen at the austenitizing temperature. This research has been conducted on a method for improving the resistance to hydrogen decomposition, which is especially accelerated in the aluminum metallic coating layer during the specific atmospheric process [4,5,6]. In addition, research on the improvement of the resistance to hydrogen delayed fractures through the control of the microstructure and the optimum alloy of ultra-high-strength steel has been actively conducted [7,8,9].

Recently, thermal desorption spectroscopy (TDS) has received attention as the most reliable hydrogen analysis method for quantitative analysis. The TDS technique is used for the purpose of studying the behavior of hydrogen released during isothermal heating. In previous studies, the TDS technique was mentioned as the most reliable technique for hydrogen analysis [10,11]. Enomoto, M. shows the reliability of the TDS method, and its finite difference code was developed to simulate the result of TDS analysis from the steel plate with martensite (or ferrite) and austenite phases [12]. Therefore, by utilizing the TDS technique, it is possible to measure the hydrogen content in the steel and to distinguish reversible or irreversible hydrogen by verifying the temperature range of hydrogen desorption. The hydrogen trap site in the steel can be characterized by its binding energy in each microstructure [12,13,14]. In the case of a diffusible hydrogen, the hydrogen traps were characterized by a reversibility that is relevant to a binding energy lower than the value of 30 kJ mol^−^^1^, which generally corresponds to the hydrogen being released at a low temperature. The diffusible hydrogen concentration was measured by means of a thermal desorption analyzer (TDA) at up to 300 °C. When the binding energy with hydrogen is above 60 kJ mol^−^^1^, these hydrogen traps are regarded as irreversible hydrogen. This irreversible hydrogen, which corresponds to the particles that form a high tensile stress between the microstructures, is considered as stable hydrogen. The activation energy of the hydrogen that is thus measured depends on the level of consistency [15]. Eventually, the behavior of these hydrogen traps can be elucidated by utilizing the TDS analysis. 

Therefore, in this study, the aims are to investigate the hot stamping process for industrial vehicles considering the industrial aspects, and to elucidate the absorption behavior of hydrogen by controlling the holding time at a specific dew point in an atmosphere source in the heating furnace. In addition, an automotive sequential process was carried out for the drying of paints after a phosphate process and an electro-deposition process, as a post-process for automotive parts. There is little literature about the hydrogen behavior during or after the phosphatizing step of the electrodeposition process on aluminum-coated hot-stamped boron steel. The present study investigates the behavior of the diffusible hydrogen of two kinds of grade steel plates that are aluminum-coated, which have tensile strengths of 1.5 GPa and 1.8 GPa, and were studied with different carbon contents in the specific hot stamping conditions. For this purpose, the TDS technique is mainly used. First, the microstructure and absorption of diffusible hydrogen were investigated depending on the soaking time in the furnace. Second, the hydrogen diffusion behavior, including automotive sequential manufacturing processing, was taken into consideration regarding hot-stamped automotive components.

## 2. Materials and Methods

### 2.1. Materials

The chemical compositions of the cold rolled steel sheets with added boron (B) on hot stamping are displayed in Table 1. The steel sheets have been distinguished into two types of alloys based on the different C content and tensile strength. First, they were hot rolled to a thickness of 3.0 mm and subsequently cold rolled to produce a cold rolled steel sheet that has a thickness of approximately 1.0 mm. Each sample was coated with Al-Si at a coating mass of 80 g/m^2^ through a continuous plating process. The Al-Si plating was conducted in order to prevent oxidation occurring on the surface of the steel sheet during austenitiation at 1173 K or higher, while providing corrosion resistance. Predominantly, aluminum plating has been reported to improve corrosion resistance by imparting excellent passivation properties. Conventionally, the chemical composition of the Al-Si plating layer consists of Al, Si, and Fe at 10:3:87. The plating thickness was 20 µm or less and the cold rolled material had a ferrite–perlite microstructure with a tensile strength of 600 MPa.

### 2.2. Simulation of the Hot Press Forming Procedure 

**Figure 1:**
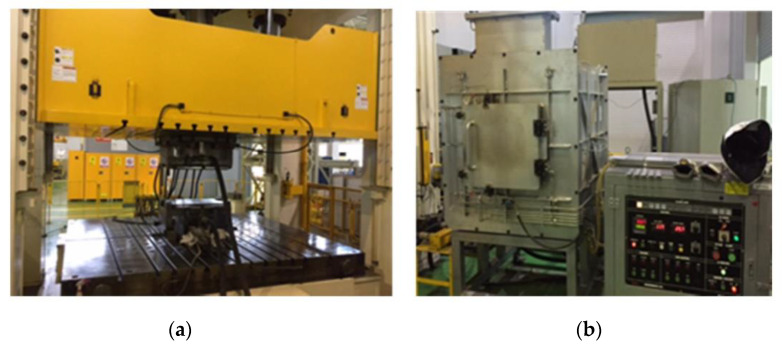
The equipment for simulating the hot stamping procedure: (**a**) the die quenching and (**b**) the heating furnace with control of the atmosphere condition.

In general, the carbon content in low-carbon steel determines its mechanical property in martensitic steel. The hot stamping steel was rapidly quenched above the annealing temperature to produce a full martensite structure that exhibits a tensile strength of 1.5 GPa and 1.8 GPa grade steels, which have different carbon contents in chemical compositions shown above in Table 1. Depending on the type of alloy design, the prior austenite produced at high temperature rapidly cools in the mold to form martensite. The holding time for the two steel grades was maintained at 150, 300, and 450 s for a hot stamping heating furnace at 1203 K to reflect the differences in the hot stamping process while maintaining various holding times. Figure 1 shows hot stamping process (made by HYUNDAI ROTEM cooperation, Uiwang-si, Korea) and experimental simulation equipment (made by SINSUNG cooperation, Ansan-si, Korea). As shown in Figure 1a, the die quenching using a metallic mold was performed. Additionally, the moisture in the atmosphere of the austenite heat treatment process in the furnace was adjusted to a dew point (D.P.) of +10 K to stimulate hydrogen inflow during the hot stamping process, as shown in Figure 1b. The flat plate sample for the production of hot-stamped steel sheet is 300 mm × 300 mm cold rolled steel sheet with a thickness of 1.0 mm, heated at a heating rate of approximately 10 K·s^−^^1^ in a heating furnace at 1203 K, and maintained until the corresponding time, with a transfer time of 9 to 13 s. After hot forming, the plate press mold was cooled to 35 K·s^−^^1^. In the case of a hot stamping steel sheet, it is also reported to have anisotropy. In this study, a tensile test piece was taken in the direction perpendicular to the rolling direction, and, in the case of a hydrogen amount analysis sample, a sample was taken from the central part of the steel sheet and analyzed and evaluated. After hot forming, the plate press mold was cooled at 35 K s^−^^1^. Hot-stamped steel sheets have been reported to have anisotropy. Consequently, a tensile test piece perpendicular to the rolling direction was analyzed. Furthermore, a sample from the center of the steel sheet was analyzed to evaluate the hydrogen content in the sample.

### 2.3. Analysis of Mechanical Property and Its Microstructure on Hot-Stamped Steels

After hot stamping process, the mechanical property was evaluated. The mechanical property was performed using the Zwick (ZwickRoell Z600 model, Fürstenfeld, Austria). The Vickers hardness of all specimens was estimated using the CLEMEX (MMT-X7 model, Quebec, Canada). In addition, in order to characterize the martensitic phases after hot stamping process, the specimens were etched using a Nital solution. The observation of martensite phase was performed using FE-SEM equipment by Carl Zeiss (8UPRA40 model, Oberkochen, Germany). The XRD analysis of samples was performed by X-ray diffraction equipment by Rigaku (DMAX model, Austin, TX, US). The metallic coating observation with analysis of chemical composition was used by FE-SEM attached with EDS equipment by Carl Zeiss (8UPRA40 model, Oberkochen, Germany). In addition, for observing fine microstructure, the TEM analysis was conducted using FEI (TECNAI G2 F20 8-TWIN model, Austin, TX, US).

For observation of the prior austenite of the martensitic microstructure, the corrosive etching was performed in a solution including 4 g of picric acid and 100 mL of distilled water, together with a few drops of hydrochloric acid at 90 °C. This rendered it possible to observe the prior austenite grain boundaries. The optical microscopy for observation of the PAGS was used by LEICA (MZ8 model, San Diego, CA, US), and the image analyzer was used for calculating the average of primary austenite grain sizes on the images with x 500 magnitude acquired from optical microscopy on all studied specimens.

### 2.4. Simulation of Automotive Sequential Lines

The body-in-white of vehicle undergoes a process for the pre-treatment of the surface protective layer. The electrophoretic deposition technique is commonly employed in the automotive painting process to establish coatings for complex metallic components with excellent corrosion resistance and covering. Laboratory scale simulations of automotive sequential lines were performed. The treatment conditions of each stage are summarized in Table 2. The sequential line consists of the zinc phosphating and electrodeposition stages, along with repeated rinsing and cleaning processes. The sequential line processes can be further categorized into the: (1) zinc-phosphating process, (2) electrodeposition process, and (3) baking process. The hydrogen absorption and desorption behavior were investigated in each of these crucial processes for coatings. The phosphating process consists of an immersion process in a chemical solution (provided by Nipsea product) that has a relatively low pH. The electrodeposition process implements the use of direct current to a metal part immersed in a belt of oppositely charged paint articles (provided by Noroo production). Following the sequential pre-treatment processes, the paint articles are attracted to the metal part and deposited to produce an even continuous film over the complete surface until the coating attains the desired target thickness of polymer coating. Electrodeposition involves immersing the metal part in a water-based solution containing the paint emulsion while applying cathodic current. The baking process was performed at 443 K for 20 min in the baking oven. Finally, the interior of the metal part was coated with a polymer coating, completing the electrodeposition sequential process.

### 2.5. Hydrogen Analysis

A 20 mm × 20 mm sample with a thickness of 1 mm was placed in a heating chamber and uniformly heated using an infrared heating radiation (IR) method at 20 K min until a final temperature of 773 K was reached. Hydrogen released during the uniform heating was detected by quadrupole mass spectroscopy. The elevated temperature was recorded by several thermocouples located on the specimen surface. The released hydrogen atoms were detected by quadrupole mass spectroscopy made by PFEIPPER VACUUM company (Aßlar in Lahn-Dill-Kreis, Germany). All samples were washed with ethanol and air dried. The hydrogen desorption curve derived from the quadrupole mass spectroscopy illustrates the molecular weight of hydrogen over time as a function of temperature. The reversible hydrogen content was quantified during the heating process by calculating the accumulated content by integrating the signal from room temperature to 553 K, corresponding to the first peak of the emission curve. Hydrogen content analysis provided retention times for various steel types. Additionally, the activation energy (E_a_) of the reversible hydrogen in two kinds of steels with different C contents and mechanical properties was evaluated through TDS by changing the heating rate to 5, 10, and 20 K min^−^^1^, respectively. Notably, the effect of automotive pre-treatment, electrodeposition coating, and the baking process was considered for the hydrogen content analysis of the aluminum-plated steel type with a tensile strength of 1.8 GPa. 

## 3. Results

### 3.1. Microstructure and Mechanical Properties

**Figure 2:**
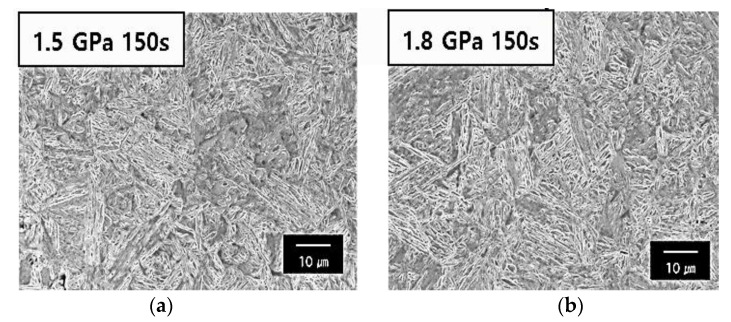
SEM images of martensitic phase: (**a**) the 1.5 GPa grade steel austenitized for 150 s at 1203 K and (**b**) the 1.8 GPa grade steel austenitized for 150 s at 1203 K.

Table 3 displays the mechanical properties obtained from the uniaxial tensile test. The hardness analysis of the various steel types shows that the hot stamping furnace has a constant value regardless of the holding time, which could be because of the difference in the C solution strengthening on the martensite as a result of the difference in the C content between the 1.5 GPa steel and 1.8 GPa steel. As shown in Table 3, in the case of the samples with a 0.23 C content, an ultimate tensile strength of approximately 1500 MPa, a yield strength of approximately 1000 MPa, and an elongation at a fracture of 8% can be obtained in three types of samples. This sample is called 1.5 GPa steel. In the case of the samples with a 0.30 C content, an ultimate tensile strength of approximately 1800 MPa, a yield strength of approximately 1000 MPa, and an elongation at a fracture of 7% can be obtained in three types of samples. This sample is termed 1.8 Gpa. The difference in the mechanical properties of the specimens for various holding times for each steel type was not observed. Therefore, this indicates that the hardness of the material is independent of the holding time in the furnace. As exhibited in Figure 2, the martensitic microstructure and the reverse transformation to the austenite phase was adequately completed for each steel type at a retention time of 150 s at 1.0 t thickness. This produced the final full martensitic structure at room temperature.

**Figure 3:**
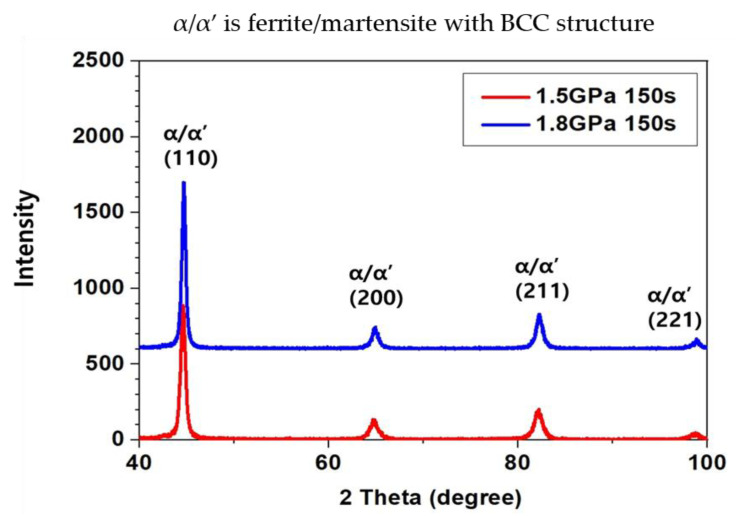
Results of X-ray diffraction of martensitic phase on the 1.5 GPa and 1.8 GPa grade steels austenitized for 150 s at 1203 K; The indication of α/α’ means ferrite/martensite with body centered (bcc) structure.

The X-ray diffraction results displayed in Figure 3 confirmed that there was an absence of a residual austenite phase. The X-ray diffraction results confirmed that both the 1.5 and 1.8 GPa grade steels produced by the martensite had a bcc crystal structure with peaks at (110), (200), (211), and (221), within the retention time of 150 s. This was the longest time taken by the hot-stamped microstructure to form martensite. Based on this result, we can assume that the specimens with holding times of 300 and 450 s also produced the fully martensite phase.

**Figure 4:**
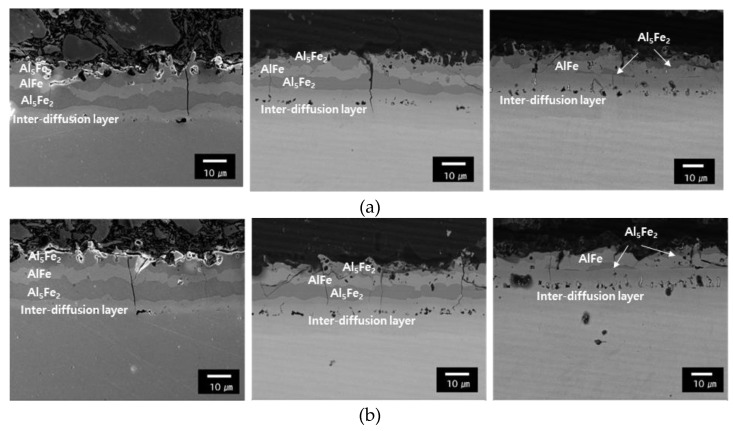
SEM images of coating: (**a**) the 1.5 GPa grade steel austenitized during 150 s, 300 s, and 450 s and (**b**) the 1.8 GPa grade steel austenitized during 150 s, 300 s, and 450 s.

Figure 4 displays the chemical composition of the coating layer for each steel type corresponding to the holding time in the furnace at 1203 K. During the initial heating stage conducted for 150 s, the plating layer consisted of four inter-layers separated by an Al-Si-Fe diffusion layer. They include a superficial layer (Al_5_Fe_2_), intermetallic layer (AlFe), intermediate layer (Al_5_Fe_2_), and inter-diffusion layer [16]. As highlighted in Figure 3a,b, the growth of the AlFe intermetallic layer due to the diffusion of Fe into the surface layer increases with the increase in the holding time of the heating furnace from 150 to 450 s for both the 1.5 and 1.8 GPa grade steel plates. The inter-diffusion layer grows in the direction where Al diffusion occurs in the base material. Table 4 displays the Fe, Al, and Si content of each layer using SEM-EDS. This demonstrates that the Fe, Al, and Si components present in each phase have similar component ratios regardless of the steel type and holding time.

**Figure 5:**
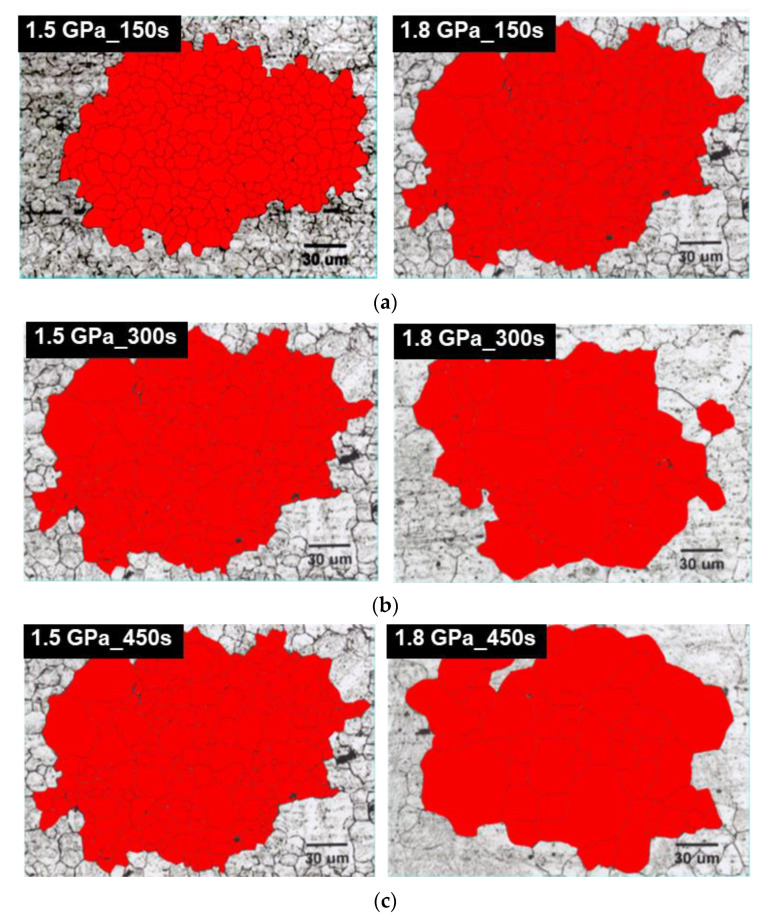
The OM images showing prior austenite grains in both 1.5 GPa grade and 1.8 GPa grade steels austenitized at 1203 K for (**a**) 150 s, (**b**) 300 s, and (**c**) 450 s.

Figure 5 illustrates the results of the prior austenite grain size (PAGS) measurements of the samples of different steel grades and holding times. The red color corresponds to the drawn grain morphology of the primary grain in each sample, and these red-colored colonies of grains in each sample are recognized for calculating the average of PAGS, as shown in Table 5. The PAGS increases with the increase in retention time for each steel type. As indicated in Table 5, the sensitivity was found to increase with an increasing retention time for the 1.8 GPa steel, but not for the 1.5 GPa steel. The austenizing temperature, which is thermodynamically dependent on an increase in the C content, was found at a lower temperature for the same hot stamping temperature of 1203 K. The 1.8 GPa grade steel under the same process conditions at each holding time was found to be faster, suggesting the initiation of aging. The PAGS measurement suggests that the 1.5 GPa grade steel was coarser than the 1.8 GPa grade steel for samples that have received the same processing. This depicts that the difference in the grain size increases with the increase in the tendency of coarsening for each heating furnace holding time, for each steel type. It has been reported that the larger the PAGS size, the lesser the hydrogen delayed fracture properties [17]. Consequently, the larger PAGS of martensite results in a higher concentration of hydrogen per unit length. This leads to the critical hydrogen concentration required for the fracture being reached faster, ultimately resulting in a hydrogen delayed fracture [17,18].

### 3.2. Hydrogen Behavior during Hot Press Forming

**Figure 6:**
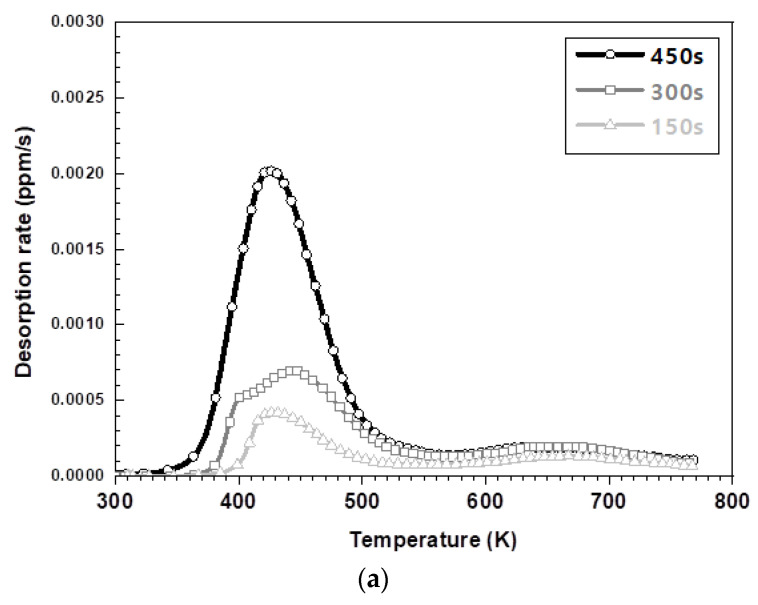

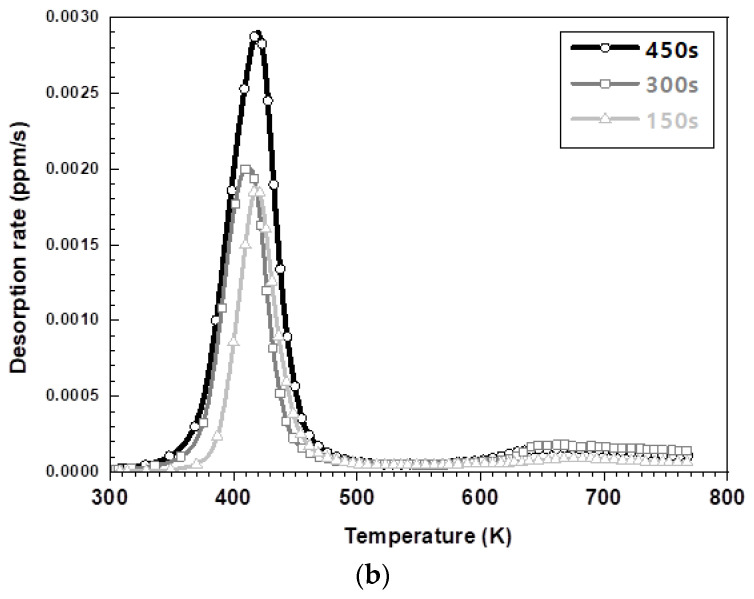
The TDS curves depending on keeping time in furnace: (**a**) the desorption curve in the 1.5 GPa grade and (**b**) the desorption curve in the 1.8 GPa grade.

Figure 6 exhibits the results of the hydrogen amount analysis immediately after hot stamping for various austenitization holding times of each steel type. Irrespective of the steel type, the total amount of hydrogen, specifically the amount of diffusible hydrogen, increased with an increasing holding time. The amount of non-diffusive hydrogen in the 573 K to 773 K range remains nearly constant because of the greater hydrogen storage on the surface for longer austenizing times. The amount of hydrogen on the surface increased due to the decomposition–recombination reaction of the water vapor in the furnace atmosphere. Figure 6a,b illustrate the lower peak temperature at which hydrogen evolution is the maximum for the 1.5 GPa grade steel compared to the 1.8 GPa grade steel. It has been reported that hydrogen is adsorbed through the surface reaction of aluminum with the moisture in the furnace atmosphere, whose mechanism is described in Equations (1)–(4) [19,20,21,22] Aluminum in the surface layer acts as a catalyst and promotes the decomposition of moisture at the surface, which introduces hydrogen through an oxidation reaction above 873 K. However, when cooled to room temperature, this aluminum layer acts as a membrane with a very low hydrogen diffusion rate, preventing the release of hydrogen, thereby trapping it [19,20,21,22].

2Al + 6H_2_O^−^ → 2 Al (OH)_3_ + 6H^+^ (1)

2Al + 4H_2_O → 2 AlO (OH) + 6H^+^ (2)

2Al + 4H_2_O → 2 Al2O_3_ + 6H^+^ (3)

Si + 2H_2_O → SiO_2_ + 6H^+^ (4)

### 3.3. Activation Energy for Various Steel Types

The TDS analysis demonstrates the difference in the hydrogen release temperature for different steel types. This is a result of the difference in the activation energy of diffused hydrogen for various steel types. The TDS analysis was conducted at 5, 10, and 20 K min^−1^ and the hydrogen diffusion behavior and the resulting peak temperature were derived through Equation (5). The Ea was calculated through the following equation:

(5) $$\frac{\partial \left(\Phi /{T_{p}}^{2}\right)}{\partial \left(1/T_{p}\right)}=-\frac{E_{a}}{R}$$

**Figure 7:**
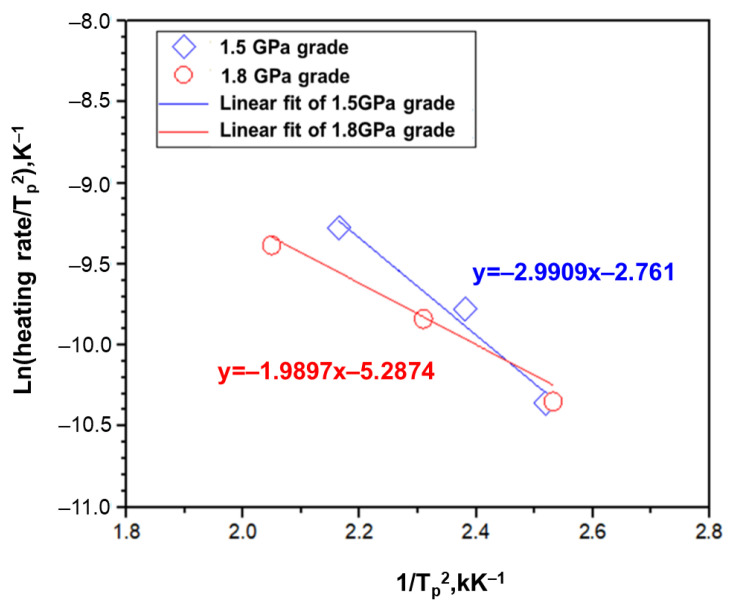
The fitting graph from the TDS results depending on the different heating rates on the 1.5 GPa grade and 1.8 GPa grade alloys.

Figure 7 displays that the fitting values of the peak temperature through TDS as a function of the rate of temperature rise for the 1.5 GPa and 1.8 GPa grade steel plates. Table 6 shows the calculated activation energy values from Figure 6. Consequently, the hydrogen diffusion energy was lower for the 1.8 GPa grade steel compared to the 1.5 GPa grade steel. This suggests that hydrogen is more easily diffused into defects or localized stress regions when a hydrogen delay fracture occurs. Therefore, 1.8 GPa grade steel can be considered more vulnerable to hydrogen delayed fractures than 1.5 GPa grade steel.

### 3.4. Behavior of Hydrogen Desorption from the Automotive Sequential Process Line

**Figure 8:**
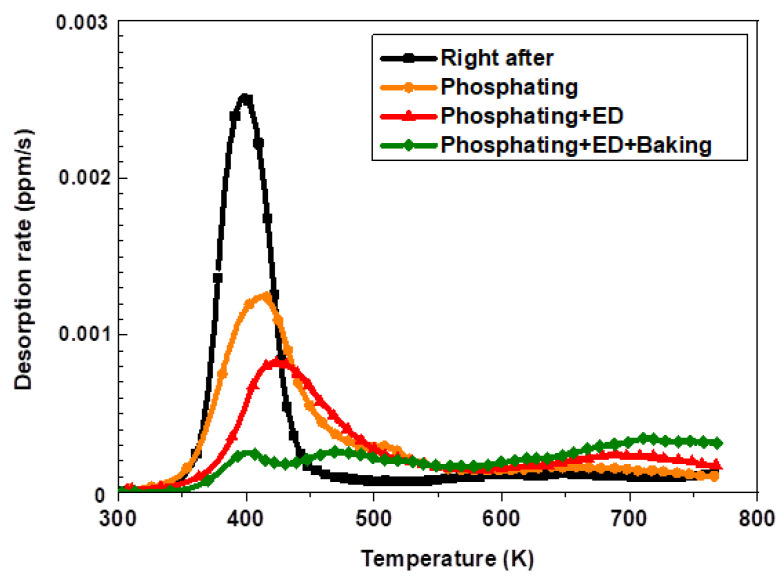
The TDS curves and behavior of diffusible hydrogen after automotive sequential process on the 300 s specimen of 1.8 GPa hot-stamped steel.

The automotive sequential process is conducted as a subsequent process for automotive parts for the drying of paints after the phosphate and electrodeposition process. The hydrogen desorption behavior was studied after each successive process in automobile manufacturing after hot stamping. First, the continuous pre-treatment and electrodeposition processes were conducted. Then, the baking heat treatment was performed for 20 min at 443 K. The amount of hydrogen was analyzed after chemically removing the polymer layer through the electrodeposition coating with a dedicated remover. Figure 8 shows the TDS graph of the hydrogen release behavior of 1.8 GPa steel after going through the automotive continuous pretreatment and the electrodeposition coating lines. The hydrogen introduced immediately after the hot stamping decreased after the final baking for each process.

**Figure 9:**
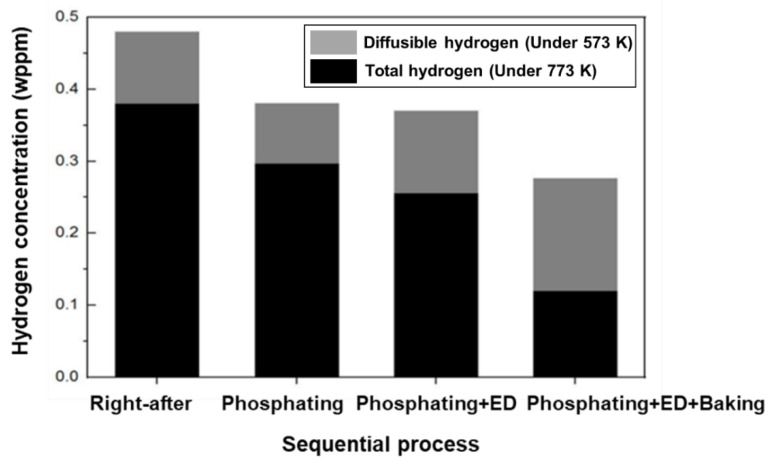
The diffusible hydrogen content after automotive sequential process on the 300 s specimen of 1.8 GPa hot-stamped boron steel.

Figure 9 depicts the results of the hydrogen analysis in each successive process of automotive manufacturing. Specifically, the amount of hydrogen introduced by the phosphate forming and electrodeposition process did not affect the additional hydrogen inflow for the hot stamping steel plate coated with aluminum. Conversely, the amount of diffusible hydrogen was found to be 0.124 wppm after the baking process, indicating that most of the hydrogen introduced during the hot stamping process was removed [6,22].

## 4. Discussion

### 4.1. Hydrogen Trapping on Steel Grades during the Hot Stamping Process

In general, the diffusible hydrogen concentration must be distinguished from theoretical concepts of lattice hydrogen C_L_ and trapped hydrogen concentrations C_T_, which are defined by their microstructural characteristics. Therefore, the diffusible hydrogen concentration is referred to in general work as 300 °C, which describes the concentration of hydrogen effusing from low carbon steel at a measurement temperature of 300 °C. On the other hand, non-diffusion hydrogen, which is characterized by being irreversible, corresponds to hydrogen released at a relatively high temperature and high temperature during TDS analysis. Accordingly, it is necessary to distinguish the diffusible hydrogen concentration, which has often been cited as the relevant part of the total hydrogen concentration in causing hydrogen embrittlement, from the lattice and trapped hydrogen concentrations. Similar to the results of this study, the diffusible hydrogen concentration integrates the hydrogen, which diffuses during isothermal heat treatment at 300 °C [23]. Therefore, the diffusible hydrogen concentration is a semi-phenomenological quantity and strongly depends on the thermal activation of trap sites [23,24], such as the sample thickness and the metallic coating with the surface treatment of the specimen. Depending on these conditions, it has to be pointed out that the diffusible hydrogen concentration cannot represent the lattice hydrogen concentration. According to TDS spectra of the complex phase (CP) steel and dual phase (DP) steel, it is obvious that the diffusible hydrogen concentration relates to the definition of the total hydrogen concentration, because no further peak, which would relate to deep trapping sites, such as precipitates or retained austenite, could be found for the investigated materials above 300 °C [23,25]. In this study, the hydrogen peaks from the TDS analysis are under 300 °C, which correspond to the diffusible hydrogen range in al-aluminum-coated martensitic steel in our previous studies [17,18,22].

The diffusion phenomenon in the furnace during hot stamping and the movement of hydrogen atoms in the internal bulk region are governed by Fick’s laws. However, the experimental results demonstrated a behavior contrary to the ideal theory. This could be due to the presence of hydrogen trapped inside various trapping sites in the microstructure, such as grain boundaries, dislocations, vacancies, and precipitates, among others [26,27,28]. These trap sites eventually influence the diffusion of hydrogen in the internal steel plate. The hydrogen is categorized into reversible and irreversible, depending on their hydrogen binding energies and relationship with their surroundings [29,30,31,32,33,34]. The equations derived from the McNabb and Foster trap model (1963), refs. [32,33] can be used to describe the hydrogen transport phenomenon, and are shown below:

(6) $$\frac{\partial C}{\partial t}=D\frac{d^{2}C}{dx^{2}}-Nr\frac{\partial v}{\partial t}-Ni\frac{\partial w}{\partial t}$$

(7) $$\frac{\partial \nu }{\partial t}=\;\mathrm{KrC}\left(1-\nu \right)-p\nu$$

(8) $$\frac{\partial \omega }{\partial t}=KiC\left(1-\omega \right)$$

where *C* is the hydrogen concentration (atoms m^−3^); *D* is the hydrogen diffusion coefficient (m^2^ s^−1^) in pure iron; *N*_r_ and *N*i are the concentrations of reversible and irreversible traps, respectively (atoms m^−3^); ν represents the occupied reversible trap fraction; w refers to the irreversible traps; *t* and *x* are the time and space variables, respectively; *K*_r_ is the trapping rate for reversible traps (m^3^ atoms^−1^ s^−1^); *K*_i_ is the trapping rate for irreversible traps; and p is the release rate for reversible traps. For different microstructures, the hydrogen diffusion could be influenced by the trapping sites.

**Figure 10:**
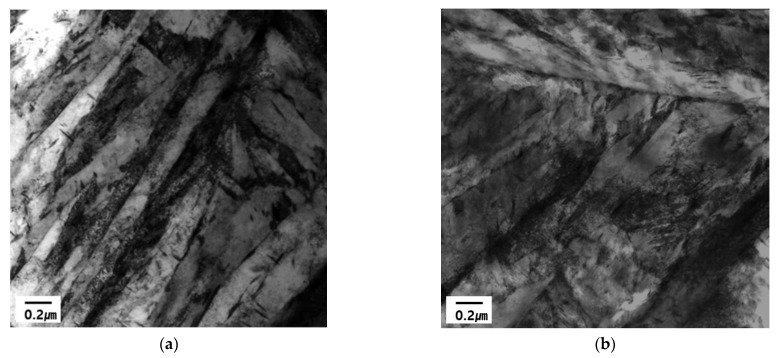
TEM image of hot-stamped boron steel: (**a**) 1.5 GPa and (**b**) 1.8 GPa grade specimens.

In previous studies, the change in the hydrogen trapping efficiency in iron introduced through grain refinement or cold work has been shown to depend on the concentration of lattice defects and their interaction energies with hydrogen [29,30]. In this study, the cold work was accounted for by altering the dislocation density, thereby increasing the density of the trapping sites. Furthermore, some non-equilibrium defects were removed by annealing as a function of the temperature and time. Two- or three-dimensional lattice defects, such as dislocations and grain boundaries, have a two-fold effect on hydrogen diffusion. These defects enhance the diffusion along the disturbed lattice regions, but also display a hydrogen trapping effect. As the grain size decreases, or the dislocation density increases, the mobility of hydrogen will increase with an increasing pipe diffusion area [29]. As the trapping mechanisms and trapping at different sites are not clearly established, the characteristic trapping energies of hydrogen (ΔE_H_) at 20.6 kJ mol^−1^ and 58.6 kJ mol^−1^ were assigned to the dislocation cores and the grain boundaries, respectively, based on the work [34]. Consequently, the discussion on the microstructure of these steels was restricted to reversible hydrogen trapping sites, which have low hydrogen binding energies. The hydrogen trapping in the cold rolled sample was dominated by weak traps, (i.e., dislocations). The hydrogen trapping at dislocation led to hydrogen-assisted crack nucleation along grain boundaries [35]. On the other hand, the side incoherent interface of the semi-coherent TiC precipitate acts like the broad semi-coherent interface when the precipitate is small. However, as the precipitate grows, it decreases the coherency of the side interface, and the desorption peak that was contributed from the side interface shifts to a higher temperature and coincides with the desorption peak of the incoherent TiC particle. The desorption activation energy associated with the side interface changes from 55.8 to 100 kJ/mol [10]. Based on previous research, calculating the activation energy with hydrogen atom on these trap sites can affect the available combinations of aluminum (coating) and steel (substrate) [6]. The coating of the surface of steel is a strongly effective parameter using the TDS technique, which means that the aluminum coating can increase the desorption temperature of hydrogen during the isothermal heating process. Eventually, the trapping energy can be estimated with slightly higher values in comparison with the non-coated steel plate. However, we mainly focus on the trapping energy of different chemical composition (C contents: 0.23 and 0.30 wt.%) specimens with similar conditions of coating in this research scope. Therefore, the chemical composition with different carbon contents can affect the eventual microstructure and significantly affect the difference in binding energies of the 0.23 wt.% C and 0.3 wt.% C alloys, as shown in Table 6. During the quenching process, the diffusion was always faster in the 1.8 GPa grade steel compared to the 1.5 GPa grade steel because of the considerable amount of carbon in the higher grade. Furthermore, a greater dislocation density generated in 0.3 wt.% C alloy results in the reduction in the total trapping energy because of the increase in the dislocation fraction and decrease in the prior grain boundary density with more coarsened grains. As highlighted in Figure 5, the prior austenite grain size of the 0.3 wt.% C alloy was coarser, indicating that the mean length of the grain boundary was less occupied in the matrix compared to the 0.23 wt.% C alloy. In general, it is difficult to demonstrate the presence of solute carbon and the dislocation density in terms of an observation of microscopic techniques. Figure 10 shows the TEM images of the martensitic phase from the 1.5 GPa and 1.8 GPa specimens during 300 s soaking time. Both samples show the typical lath martensitic structure, as shown in Figure 10. However, it is difficult to observe a meaningful difference depending on the carbon contents shown in Figure 10b compared with the as-received specimen shown in Figure 10a. In the case of the martensitic phase, it is a general phenomenon that the high density of dislocation is dispersed in a lattice structure with a body-centered tetragonal (BCT). Ultimately, it can be approximately assumed that the reason for lower trapping energies in 1.8 GPa grade steel is due to the competitive occupation of hydrogen in the dislocation and grain boundary microstructures.

### 4.2. Hydrogen Desorption Behavior of during Automotive Process of Hot-Stamped Parts

The noticeable decrease in the hydrogen peaks in the TDS curves of the hot-stamped specimens after the phosphating process confirmed the result in this study. The diffusible hydrogen peak in the TDS curve diminished after the electrodeposition process. The aluminum coating was shown to suppress additional hydrogen uptake during zinc phosphating. The operating temperature of the hot stamping process induces a desorption of trapped hydrogen in the steel, as shown in Table 7. Previous studies indicate that the major hydrogen uptake was initiated during or after the phosphatizing phase of the electrodeposition process on cold rolled steel plates [36]. The diffusible hydrogen inflow in the automotive line suggested that the majority of the hydrogen desorption occurs during the phosphatizing phase of the electrodeposition process for conventional cold-formed components. A substantial amount of hydrogen is generated during the formation of phosphating crystals. Additionally, hydrogen is electrochemically adsorbed on the surface layer during the reactions, and is then absorbed into the steel through sequential diffusion, driven by a hydrogen concentration gradient [37]. However, thin alumina coatings have been reported to be an effective hydrogen diffusion barrier, significantly reducing the permeation of hydrogen into steel [38]. Notably, the aluminum coating using aluminum oxide prevents hydrogen absorption because of its low hydrogen diffusivity and solubility at room temperature, as emphasized by the results of the diffusible hydrogen analysis after the automotive sequential process.

## 5. Conclusions

This study investigated the hydrogen uptake and desorption behavior of the hot stamping process and automotive pre-treatment line on aluminized low carbon steel. The present study focused on diffusible hydrogen in aluminum–silicon-coated hot-stamped boron steel, both sequentially during hot press forming process and in pre-treatment sequential lines of the automotive manufacturing process. The conclusions drawn from the experimental results are presented as follows:

- First of all, in the hot stamping procedure, as the soaking time increases in the heating furnace at a specific dew point, the more diffusible hydrogen is absorbed into the aluminum–silicon-coated hot-stamped boron steel parts;
- In addition, regarding the microstructure, the increase in soaking time causes a coarsening of the prior austenite grain on the fully martensitic steel, and the inter-diffusion layer grows in the direction where Al diffusion occurs in the base material in the heating furnace during the hot stamping process;
- Based on the TDS analysis of hydrogen absorbed from hot stamping, the activation energy of diffusible hydrogen in 1.8 GPa grade steel is a low value compared with that of 1.5 GPa grade steel. This means that the tendency of diffusion is more susceptible to vulnerable defect sites at a high level of tensile strength grade;
- In the sequential automotive manufacturing process, the additional hydrogen did not flow into the surface, and an electro-deposition process, including baking, was effective in removing diffusible hydrogen atoms up to the hydrogen content level of the as-received state on the aluminum–silicon-coated hot-stamped parts;
- Based on those results, the hydrogen absorption was facilitated during hot press forming, but the hydrogen was sequentially desorbed during automotive sequential lines on aluminum-coated hot-stamped steel parts.

## Figures and Tables

**Table 1:** Chemical compositions of studied steels.

Specimen	C	Si + Mn + Cr	Ti + Nb	B	Fe
1.5 GPa steel	0.23	1.8	0.05	0.002	Bal.
1.8 GPa steel	0.30	1.8	0.05	0.002	Bal.

**Table 2:** The pre-treatment condition of automotive sequential line.

Automotive Process	Production	pH	Temp, (K)	Time, (s)	Etc
Zinc-Phosphating	Nipsea	pH 3.12	328	180	
Electrodeposition	Noroo		293	300	178 V
Baking condition			433	1200	

**Table 3:** The values of parameter from strain–stress curves.

Specimen	Soaking Time (s)	YP (MPa)	TS (MPa)	El. (%)	Vickers Hardness (Hv)
1.5 GPa-	150	1023	1589	8.0	479
	300	1021	1602	8.1	481
	450	1028	1599	8.5	477
1.8 GPa-	150	1211	1808	7.5	572
	300	1205	1810	7.7	570
	450	1220	1811	8.0	574

**Table 4:** Chemical composition of Al-Si coating as a function of austenitizing time at 1203 K in 1.5 GPa grade and 1.8 GPa grade steels.

Specimen	Soaking Time (s)	Superficial Layer (Al_5_Fe_2_)			Intermetallic Layer (AlFe)			Intermediate Layer (Al_5_Fe_2_)			Inter-Diffusion Layer		
		Al	Fe	Si	Al	Fe	Si	Al	Fe	Si	Al	Fe	Si
1.5 GPa-	150	50.9	46.8	2.3	27.8	64.0	8.2	51.8	46.2	2.0	12.7	81.9	5.4
	300	52.6	47.2	0.2	30.2	62.8	7.0	50.2	48.8	1.0	15.5	79.0	5.5
	450	53.0	46.4	0.6	31.1	61.7	7.2	49.8	49.1	1.1	17.9	76.1	6.0
1.8 GPa-	150	51.8	46.2	2	28	63.7	8.3	52	45.8	2.2	11.8	82.3	5.9
	300	52.4	47.1	0.5	30.4	62.5	7.1	49.9	49	1.1	15.9	78.3	5.8
	450	53.4	46.3	0.3	32.4	64.2	7.4	49.9	48.8	1.3	18.5	75.3	6.2

**Table 5:** Variations of prior austenite grain size as a function of austenitizing time at 1203 K in 1.5 GPa grade and 1.8 GPa grade steels.

Specimen	150 s	300 s	450 s
1.5 GPa grade	10.80 μm	13.15 μm	13.30 μm
1.8 GPa grade	11.80 μm	21.03 μm	25.90 μm

**Table 6:** Activation energy of diffusible hydrogen under 573 K in the studied alloys.

Specimen	Activation E from TDS Analysis
1.5 GPa grade	24.01 kJ mol^−1^
1.8 GPa grade	16.15 kJ mol^−1^

**Table 7:** Diffusible hydrogen and total hydrogen from TDS results after sequential process.

Specimen	Diffusible H (wppm)	Total H (wppm)
Right after hot stamping	0.392	0.482
Phosphating	0.295	0.390
Phosphating + Electro-deposition	0.249	0.383
Phosphating + Electro-deposition + Baking	0.124	0.267

## Data Availability

The data presented in this study are available on request from the corresponding author.
